# Environmental controls of mangrove propagule and seedling establishment: salinity, hypoxia, sulfide, sediment instability, and early root function

**DOI:** 10.3389/fpls.2026.1986366

**Published:** 2026-10-21

**Authors:** Min Zhang, Huanshi Zhang, Abdul Khalique, Zameer Hussain, Bilquees Bozdar, Muzammil Hussain, Fred Wang-Fat Lee, Steven Jing-Liang Xu, Min Pan, Tsz Wai Ng, Tao Lang, Nazir Ahmed

**Affiliations:** 1 College of Horticulture, Jinling Institute of Technology, Nanjing, China; 2 Faculty of Crop Production, Sindh Agriculture University, Tandojam, Pakistan; 3 College of Agriculture, Institute of Rice Industry Technology Research, Key Laboratory of Plant Resource, Guizhou University, Guiyang, Guizhou, China; 4 Guangxi Key Laboratory of Biology for Mango, College of Agriculture and Food Engineering, Baise University, Baise, Guangxi, China; 5 School of Science and Technology, Hong Kong Metropolitan University, Hong Kong, Hong Kong SAR, China; 6 Hong Kong Metropolitan University Shenzhen Research Institute, Shenzhen, China

**Keywords:** mangrove establishment, porewater salinity, root function, seedling establishment, sulfide exposure

## Abstract

Mangrove propagules and seedlings encounter interacting environmental constraints before secure anchorage and functional root systems develop. This narrative review synthesizes hydrological, physiological, biogeochemical, and sediment evidence to organize establishment into propagule delivery, retention, rooting, seedling persistence, and progression toward sapling recruitment. Tidal connectivity, elevation, hydroperiod, and drainage influence both propagule movement and root-zone conditions. Porewater salinity imposes osmotic and ionic demands, whereas prolonged oxygen limitation and sulfide exposure can impair root metabolism. Erosion, burial, and hydrodynamic disturbance can prevent establishment before physiological tolerance becomes decisive. Species, developmental stage, microsite conditions, and biotic interactions modify these responses. The synthesis supports three conclusions: propagule supply must be distinguished from post-arrival loss; similar mortality patterns can arise from different mechanisms; and successful establishment requires persistence across successive physical and physiological constraints. We therefore propose a diagnosis-first framework connecting field cues to confirmation measurements, competing explanations, and conditional restoration actions. The framework is an evidence-synthesized conceptual guide, not a validated predictive model or a source of universal thresholds. Its practical implication is to assess limiting site processes before committing to planting, while retaining assisted regeneration where propagule supply is demonstrably inadequate. Multi-site, stage-resolved monitoring is needed to test whether this approach improves restoration outcomes across geomorphic settings and disturbance histories.

## Graphical abstract

**Figure:**
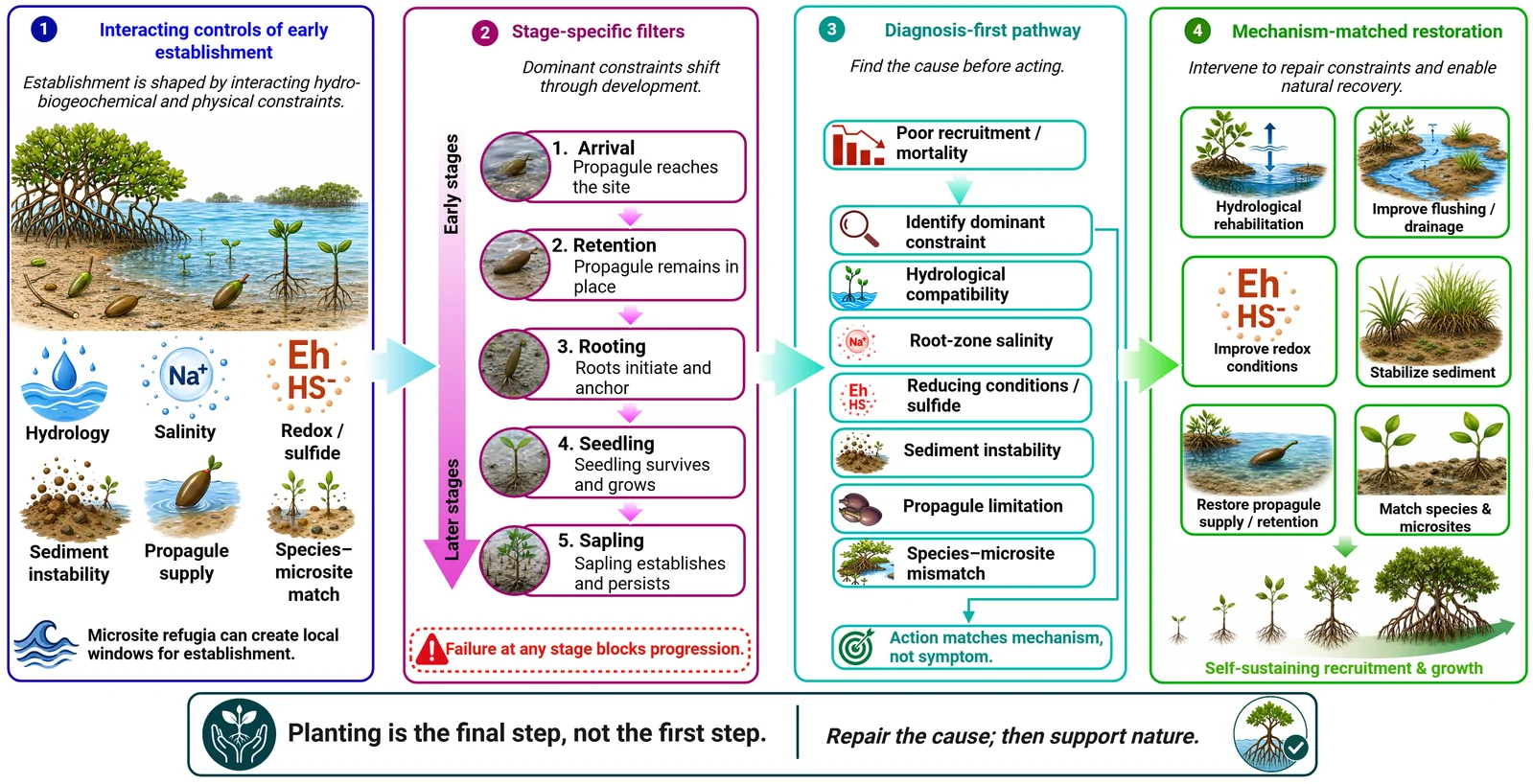

## 1 Introduction

Mangrove establishment involves successive transitions from propagule delivery and retention to root initiation, seedling persistence, and sapling recruitment. Field and experimental studies identify distinct bottlenecks in propagule supply, low-disturbance rooting opportunities, and subsequent anchorage (Sousa et al., 2007; Balke et al., 2011; Kibler et al., 2022). Salinity tolerance and waterlogging adaptations therefore do not make every intertidal site suitable for every species or developmental stage (Krauss et al., 2008; Kodikara et al., 2018). Because loss may occur before rooting or after seedling emergence, visible mortality alone cannot identify the failed transition.

Mangrove restoration seeks recovery of a self-sustaining ecosystem, but planted-seedling survival is only one component of that outcome. Syntheses show that survival and ecosystem recovery vary with environmental setting, species, intervention, and restoration age (Su et al., 2021; Gorman et al., 2022). Ecological mangrove rehabilitation and adaptive management already emphasize assessment of site hydrology, recovery of ecosystem processes, and planting only where natural regeneration is insufficient (Lewis, 2005; Ellison et al., 2020; Zimmer et al., 2022). We use “diagnosis-first” to connect these established principles with the mechanisms and life stages at which establishment fails, rather than to claim a new general principle of ecological restoration.

Among the environmental controls governing early establishment, hydrology provides the primary physical template. Tidal connectivity, surface elevation, hydroperiod, microtopography, and drainage jointly determine propagule transport and retention, inundation exposure, water residence time, and the sediment environment experienced by developing roots (Van Loon et al., 2016). By regulating water exchange, hydrology also controls porewater salinity, oxygen availability, redox conditions, sulfide exposure, and sediment properties that ultimately influence root development and seedling persistence (Lyimo and Mushi, 2005; Pérez-Ceballos et al., 2020, 2022). These hydro-biogeochemical interactions demonstrate that early establishment is governed by coupled physical and physiological processes rather than by any single environmental variable.

Porewater salinity, reducing sediment conditions, sediment instability, propagule limitation, and microsite variability further modify establishment success, but none operates independently of hydrological context. Salinity responses vary with species identity, developmental stage, flooding regime, and exposure duration (Kodikara et al., 2018; Kimera et al., 2024; Hu et al., 2025). Likewise, erosion, burial, sediment texture, hydrodynamic disturbance, and local microsite characteristics influence whether propagules remain in place long enough to establish functional roots (Balke et al., 2011; He et al., 2022; Kibler et al., 2022; van Hespen et al., 2022). These interacting controls explain why neither a single salinity threshold nor generalized species-tolerance rankings can reliably predict restoration outcomes across sites.

Existing reviews synthesize mangrove seedling ecology, restoration interventions, and planting outcomes (Krauss et al., 2008; Gerona-Daga and Salmo, 2022; Gorman et al., 2022; Zimmer et al., 2022). Complementary primary studies resolve hydrological suitability, seedling physiology, or resistance to sediment disturbance, but at different scales and with different endpoints (Van Loon et al., 2016; Li et al., 2020; van Hespen et al., 2022). The contribution of the present review is to connect those evidence types through stage-specific diagnostic questions, explicitly separating natural recruitment from survival of transplanted seedlings and separating observed associations from proposed interventions.

This review synthesizes how hydroperiod, tidal connectivity, elevation, porewater salinity, reducing sediment conditions, sulfide exposure, sediment instability, propagule processes, species traits, and microsite variability interact during early mangrove establishment. We first organize published evidence by establishment stage and mechanism, then use this synthesis to propose field measurements and conditional restoration responses. Because establishment constraints vary among geomorphic settings, species, developmental stages, and disturbance histories, this framework is intended as a diagnostic guide for identifying likely limiting mechanisms rather than a universal decision rule. Hydrology is a useful starting assessment because it can modify several constraints simultaneously, but it need not be the dominant limitation at every site. The framework requires local testing and does not establish that diagnosis-first management outperforms alternative restoration approaches.

### 1.1 Literature synthesis approach and framework development

This is a narrative, mechanism-focused review, not a systematic review or meta-analysis. The revision uses the literature cited in the submitted manuscript as its starting corpus and supplements it with targeted web searches and checks of publisher and scholarly repository records. Searches combined mangrove with establishment or seedling and themes including hydroperiod and tidal connectivity; salinity and flooding; hypoxia, redox and sulfide; erosion, burial and anchorage; species and life stage; restoration; and vegetation competition, crab bioturbation, and plant-associated microbes. Additional searches used exact article titles to resolve attribution and bibliographic inconsistencies. Recent evidence from 2016–2025 was prioritized for redox and biotic processes, while older experiments were retained where they directly tested foundational mechanisms. No original database search log, screening counts, or prespecified protocol was available for reconstruction; consequently, exhaustive coverage and freedom from selection bias are not claimed.

Studies were retained for synthesis when they reported mangrove propagule or seedling responses, quantified a physical or biogeochemical process relevant to establishment, or evaluated restoration interventions linked to these processes. Controlled experiments inform mechanistic interpretation; field observations establish environmental context and associations; restoration case studies inform intervention feasibility. Studies of established trees or sediment microbial communities are identified as indirect evidence when extrapolated to recently rooted seedlings. Reviews provide context but are not counted as independent experimental confirmation. Studies testing mangrove-derived microbes only on crop plants were not used as direct evidence of mangrove establishment benefits.

Evidence was organized into six groups: hydrological filters; salinity filters; redox and sulfide filters; sediment stability filters; species and life-stage modifiers, including microsite and biotic interactions; and restoration interventions. Within each group, interpretation distinguishes study setting, organism and developmental stage, experimental versus observational design, response endpoint, and limits of transferability. Findings from different regions are compared as conditional mechanisms rather than pooled into global tolerance thresholds. Contrasts, such as vegetation facilitating propagule retention but suppressing seedling growth through competition, are retained rather than treated as inconsistent evidence.

**Figures 1**–**3** summarize the mechanisms and contrasts discussed in Sections 2–7; **Table 1** identifies illustrative evidence for each filter. Section 8 and **Table 2** translate those mechanisms into candidate diagnoses, confirmation measurements, alternatives, and conditional first actions. This translation is the authors’ interpretive synthesis: individual studies support components, not validation of the entire decision pathway. No formal risk-of-bias score, numerical evidence weighting, pooled effect size, or predictive calibration was applied. Figure symbols and arrows therefore express conceptual relationships only. Geographical and taxonomic imbalance, short experimental durations, variable measurement methods, and the initial citation corpus constrain the synthesis. The row-specific sources in **Table 2** make its evidence basis inspectable without implying that all listed actions were tested together.

**Figure 1:**
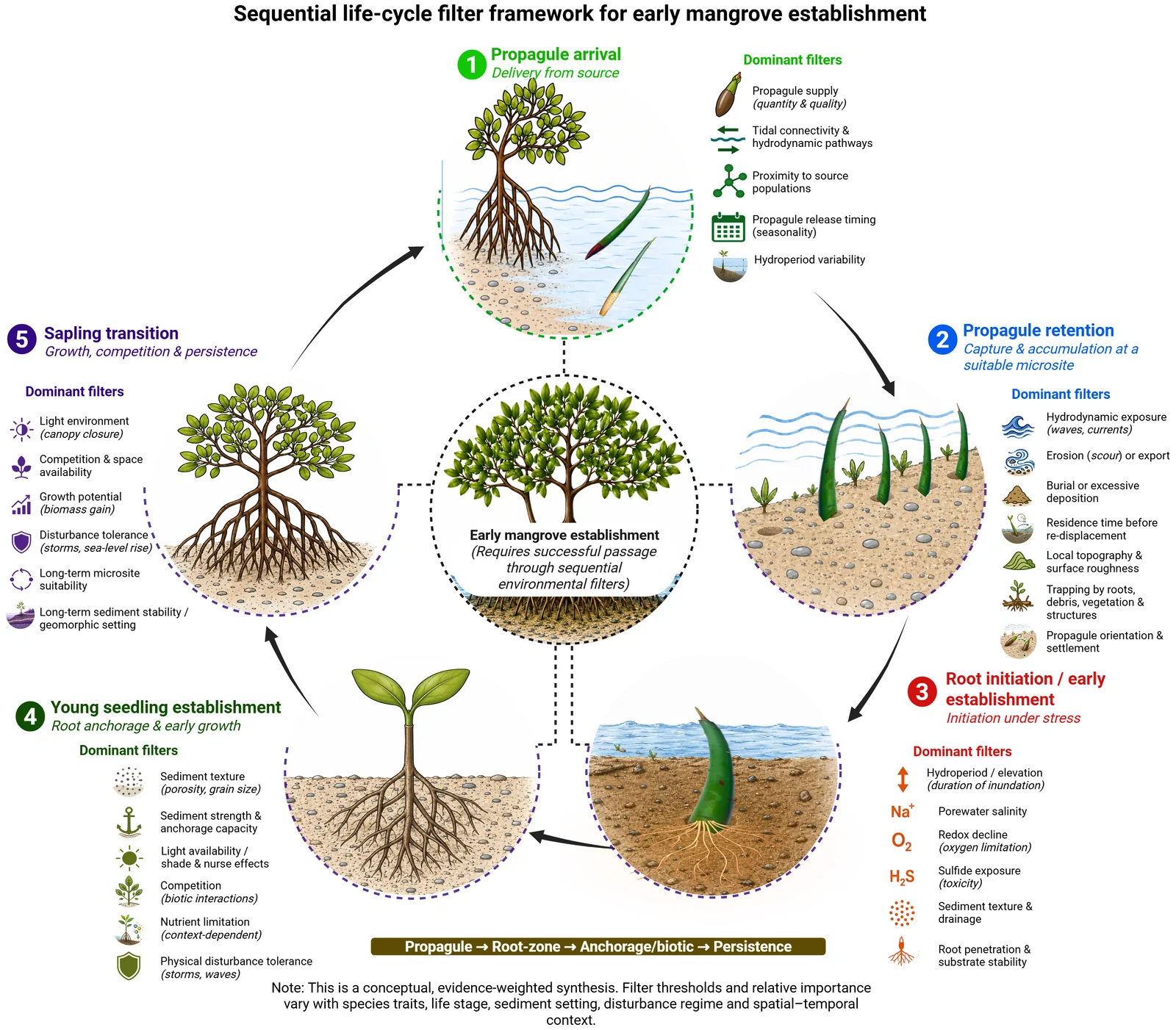
Sequential filters in early mangrove establishment. This evidence-synthesized conceptual framework integrates propagule-supply observations (Sousa et al., 2007), experimental rooting windows (Balke et al., 2011), anchorage tests (Kibler et al., 2022), and seedling-survival observations (Sloey et al., 2022), with the ecological synthesis of Krauss et al. (2008). Arrows summarize possible developmental progression; constraints overlap and can recur. For viviparous taxa, germination precedes dispersal, so the post-arrival stage refers to rooting. This is not an empirical model: no transition probability, universal threshold, fixed causal sequence, or numerical evidence weighting is implied. Components were grouped by stage using the approach described in Section 1.1.

**Figure 2:**
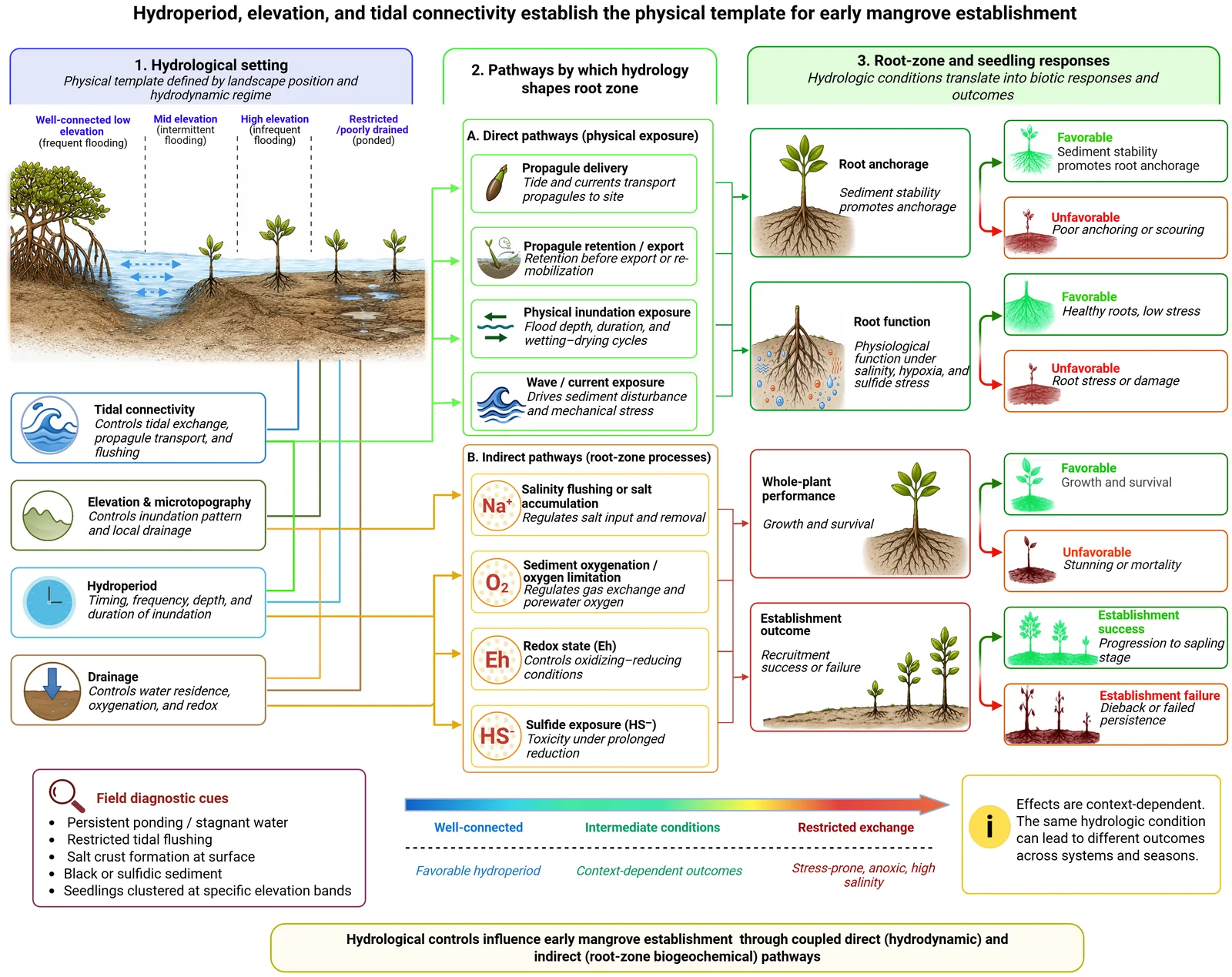
Direct and indirect links between hydrological setting and establishment. This evidence-synthesized conceptual framework combines hydrological classification (Van Loon et al., 2016), restoration observations of exchange and porewater chemistry (Pérez-Ceballos et al., 2020, 2022), seedling inundation experiments (Li et al., 2020), and anchorage studies (Kibler et al., 2022). Components were grouped by physical transport and root-zone pathways as described in Section 1.1. Arrows indicate mechanistic links, not fitted effects or fixed causal sequences. The pathways are schematic: well-connected sites can remain unsuitable because of excessive inundation or exposure, while some naturally restricted basins support mangroves. Oxygen limitation, redox potential, and dissolved sulfide are related but distinct variables; sulfide speciation depends on pH. No universal elevation or hydroperiod threshold is implied.

**Figure 3:**
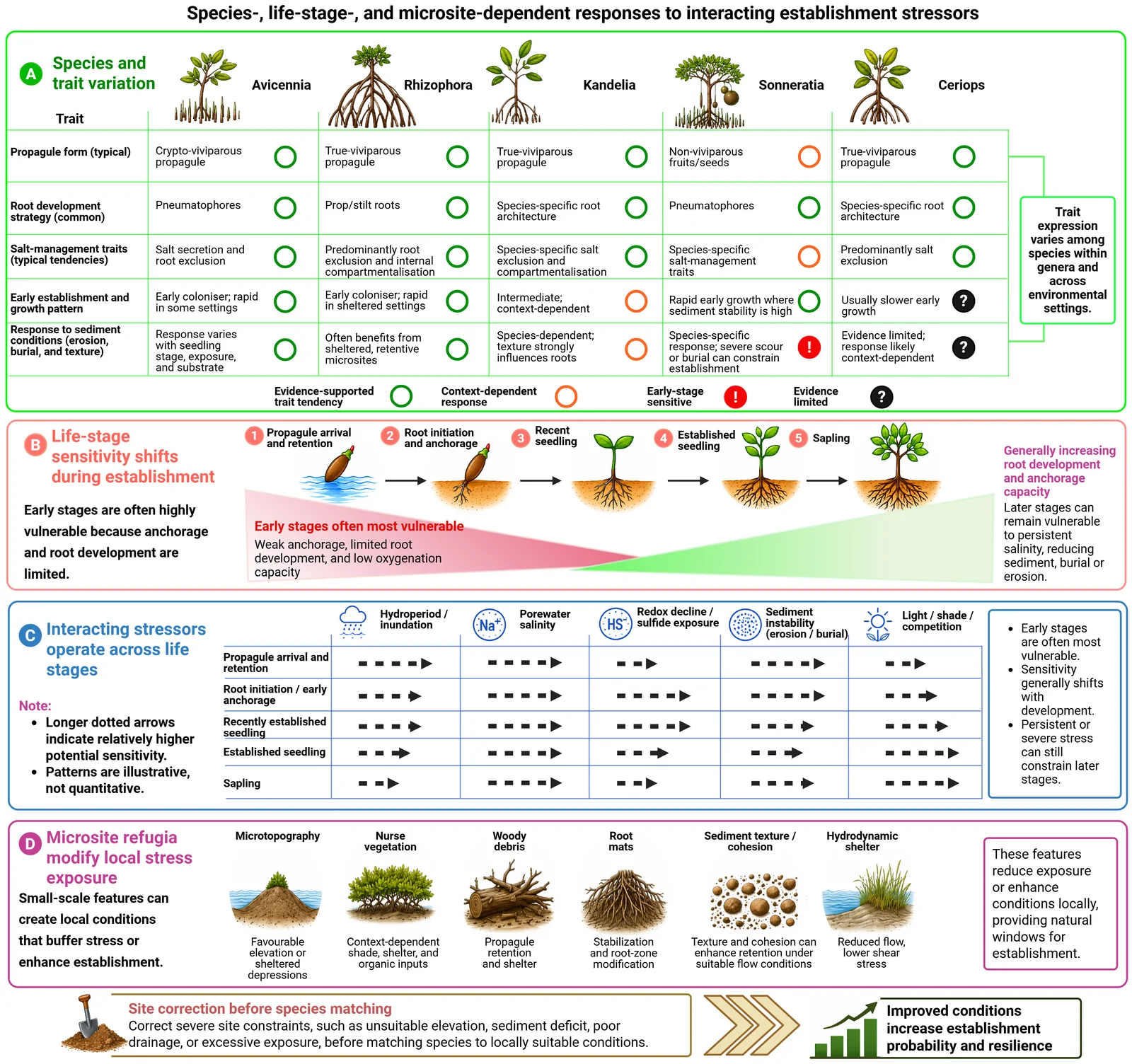
Context dependence of species, life-stage, and microsite responses. This evidence-synthesized conceptual framework presents study-linked examples rather than genus-level tolerance rankings. **(A)** draws on salinity experiments (Kimera et al., 2024), texture experiments (He et al., 2022), and cross-species sediment-disturbance experiments (van Hespen et al., 2022). **(B)** connects rooting windows, anchorage, and later seedling survival (Balke et al., 2011; Kibler et al., 2022; Sloey et al., 2022). **(C)** contrasts vegetation facilitation and competition with burrow and microbial modification (Peng et al., 2018; Booth et al., 2019; Teutli-Hernández et al., 2019; Smith et al., 2022; Subedi et al., 2022). **(D)** states limits of transfer. As described in Section 1.1, these relationships synthesize experimental and field evidence across settings; they are not an empirical model, quantified sensitivity scale, or fixed causal sequence.

**Table 1:** Mechanistic filters and illustrative evidence. Study-specific examples support the mechanisms below; they do not establish universal thresholds or validate intervention outcomes.

Filter	Measurements	Mechanistic role	Illustrative evidence	Conditional interpretation
Hydrology	Water levels; tidal datum; inundation duration and depth; drainage; connectivity.	Changes propagule transport, exposure, flushing and root-zone conditions.	Reconnection: Pérez-Ceballos et al. (2020). Inundation experiment: Li et al. (2020).	Compare hydroperiod with local reference habitat. Rehabilitate only confirmed impairment.
Salinity	Porewater salinity or EC at rooting depth; seasonality; freshwater input.	Changes water uptake, ion balance and energetic demand.	Species responses: Kimera et al. (2024). Age dependence: Kodikara et al. (2018). Interactions: Hu et al. (2025).	Distinguish persistent accumulation from seasonal exposure and other causes of stunting.
Redox and sulfide	Redox; dissolved sulfide; pH; depth; drainage; roots and organic matter.	Oxygen limitation and sulfide can impair root function; redox is not a direct oxygen measurement.	Establishment experiment: Lyimo and Mushi (2005). Root oxygen–nitrogen coupling: Cheng et al. (2020). Field degradation: (Pérez-Ceballos et al., 2022).	Dark sediment is not diagnostic. Established-root and degradation studies are indirect evidence for newly rooted seedlings.
Sediment stability	Bed-level change; scour; burial; grain size; exposure; anchorage.	Export, burial and erosion may prevent retention or remove rooted seedlings.	Texture: He et al. (2022). Anchorage: Kibler et al. (2022). Eight-species experiment: van Hespen et al. (2022).	Separate settlement loss from post-rooting removal. Interpret mechanical thresholds in local sediment and flow conditions.
Propagule supply	Source stands; viable propagule arrival; seasonal release; retention after tides.	Limits the number of individuals entering successive establishment stages.	Supply: Sousa et al. (2007). Rooting window: Balke et al. (2011). Retention: Yando et al. (2021).	Absence of seedlings does not distinguish absent supply from rapid post-arrival loss.
Species, stage and biota	Species and stage; elevation; light; competitors; crab activity; root associations.	Modifies stress exposure, retention, predation and physiological response.	Nurse effects: Teutli-Hernández et al. (2019). Light: Sloey et al. (2022). Invasion: Peng et al. (2018). Microbes: Akaji et al. (2024).	Facilitation and competition may coexist. Do not infer seedling requirements from adult zonation alone.

EC means electrical conductivity. Propagules include viviparous units and non-viviparous fruits or seeds.

**Table 2:** Field diagnostic framework for early establishment failure.

Domain and field cues	Candidate mechanism	Confirmation and alternatives	Conditional first action	Component evidence and limits
Hydrological incompatibility Ponding; blocked channels; narrow recruitment bands.	Restricted exchange, drainage impairment or elevation mismatch.	Water-level series, drainage and local tidal elevation. Compare natural basin ponding and seasonal inundation.	Where impairment is confirmed, evaluate flow-path rehabilitation or elevation compatibility before planting.	Van Loon et al. (2016); Pérez-Ceballos et al. (2020); Corona-Salto et al. (2025). Site studies; no universal engineering design.
Salinity accumulation Salt crusts; patchy mortality; stunting.	Poor flushing, evaporation, restricted freshwater supply or long residence time.	Repeated root-depth salinity/EC with hydrology. Rule out redox, drought, nutrition and species-appropriate seasonal salinity.	Consider restoring water exchange or freshwater pathways where the cause and ecological consequences are established.	(Kodikara et al., 2018); Kimera et al. (2024); Pérez-Ceballos et al. (2020). Experiments support responses, not a common field threshold.
Reducing root zone Odour; weak rooting; dieback.	Oxygen limitation or sulfide exposure under persistent saturation.	Redox, sulfide, pH, depth, season and drainage. Compare natural reduced sediment; assess salinity and physical root damage.	Consider correcting impaired exchange; avoid indiscriminate drainage or excavation of sulfidic sediment.	Lyimo and Mushi (2005); Cheng et al. (2020); Pérez-Ceballos et al. (2020); Aller et al. (2024). Mixed seedling, root and sediment evidence.
Physical instability Scour; burial; tilted or uprooted seedlings.	Sediment movement and weak anchorage; exposure may act with root impairment.	Bed-level change, burial depth, currents/waves and anchorage. Distinguish herbivory, poor rooting and exceptional storms.	Evaluate shelter, sediment-process recovery or relocation to stable microsites before repeating planting.	Balke et al. (2011, 2013b); Kibler et al. (2022); van Hespen et al. (2022). Mechanical evidence; thresholds are conditional.
Propagule limitation No seedlings; few arrivals or rapid losses.	Source isolation, interrupted dispersal, export or failed retention.	Track viable arrival and fate across release periods and tides. Rule out rapid mortality and episodic recruitment.	Protect source stands and dispersal paths; consider assisted regeneration when habitat is suitable but supply is inadequate.	Sousa et al. (2007); Yando et al. (2021); Smith et al. (2022). Supply and retention observations do not alone prove habitat suitability.
Species–microsite mismatch Species or stages differ in survival.	Mismatch with elevation, substrate, light, exposure or developmental requirements.	Compare stage-specific responses and reference recruitment. Rule out unequal arrival and unresolved chemical or physical stress.	Match species and life stage to verified microsites; reassess persistent site constraints.	He et al. (2022); Sloey et al. (2022); Hu et al. (2025). Local or experimental contrasts, not global rankings.
Biotic modifiers Dense invaders; propagule damage; burrow-associated patches.	Competition or facilitation; predation; sediment reworking; microbial feedbacks.	Measure light, vegetation identity, burrows, damage and substrate change. Pair root observations with chemistry; do not infer microbial benefit from presence alone.	Test targeted vegetation or recruitment protection measures where justified. Preserve beneficial fauna; microbial inoculation requires validation.	Peng et al. (2018); Van Nedervelde et al. (2015); Booth et al. (2019); Subedi et al. (2022); Inoue et al. (2024). Microbial compartments and life stages differ.
Persistent stunting Survival without progression.	Combined sublethal stress, competition or nutrient limitation.	Track growth with hydrology, salinity, chemistry, light and root development. Compare normal species growth and seasonal effects.	Test the leading mechanism before fertilizer addition or further planting; reassess after response monitoring.	Cheng et al. (2020); He et al. (2022); Hu et al. (2025). Integrated diagnosis is the authors’ synthesis, not a validated test.

This is an evidence-informed diagnostic aid, not a validated decision rule. Sources support mechanisms or context, not the complete row of management recommendations. Field cues are non-specific; several constraints may co-occur. Insufficient evidence is recorded as unresolved. Assess local tidal setting, reference habitat, season, species, life stage, and ecological consequences before intervention. No universal chemical thresholds, intervention order, or recovery time is specified.

## 2 Early mangrove establishment as a sequential, stage-specific process

Early mangrove establishment should not be treated as a single seedling-survival endpoint. It is a sequence of linked transitions from propagule production and delivery to retention, rooting or germination, young-seedling establishment, and persistence toward sapling recruitment. Failure at a transition can prevent progression even when later-stage conditions would otherwise be suitable (Sousa et al., 2007; Balke et al., 2011). As summarized in **Figure 1**, the dominant filters shift through the early life cycle: dispersal and propagule supply regulate arrival, hydrodynamic exposure and microsite structure regulate retention, sediment conditions influence rooting, and anchorage, competition, disturbance tolerance, and microsite stability determine persistence (Krauss et al., 2008; Balke et al., 2011). This stage-specific perspective is important for restoration because poor recruitment may originate before visible seedling mortality, including limited propagule delivery, failed retention, or unsuccessful root initiation.

The first filter is propagule availability and delivery to the restoration site. Mangrove propagules are mainly water-borne, and their dispersal is influenced by propagule traits, release timing, buoyancy, tidal currents, wind, coastal configuration, and the spatial distribution of source populations (Van der Stocken et al., 2019). Consequently, even a hydrologically rehabilitated site may recover slowly when nearby seed sources are sparse, dispersal pathways are interrupted, or propagules are transported beyond suitable settlement zones (Sousa et al., 2007). Propagule arrival, however, is only the beginning of establishment. A propagule must also remain in a suitable microsite long enough to orient, settle, and initiate roots before being exported, buried, or displaced by the next tidal or wave disturbance. Retention and post-settlement fate must therefore be measured separately from arrival (Yando et al., 2021).

Retention is therefore a distinct bottleneck between dispersal and physiological establishment. On exposed tidal flats, newly deposited propagules and recently rooted seedlings require temporary periods of sufficiently low physical disturbance to develop anchorage (Balke et al., 2011). Cross-shore gradients in waves, currents, erosion, and sediment mobility can strongly influence whether this low-disturbance window occurs (Balke et al., 2013a). Retention should therefore not be interpreted as a passive result of tidal deposition. It reflects the interaction of hydrodynamic forcing, local elevation, sediment properties, propagule morphology, surface roughness, and trapping by vegetation, roots, woody debris, or microtopographic depressions. The importance of vegetation trapping is itself conditional on tidal regime and position within the habitat (Smith et al., 2022).

Root initiation links settlement to sediment exposure, but its constraints cannot be inferred from a single substrate experiment. He et al. (2022) found species-specific root and growth responses of *Kandelia obovata* and *Sonneratia apetala* to sediment texture and nitrogen addition. Complementary experiments show that inundation changes *Avicennia marina* physiology (Li et al., 2020), while cross-species erosion and accretion treatments reveal differences in dislodgement and root development (van Hespen et al., 2022). Together, these studies separate substrate effects on root growth, flooding effects on physiological function, and sediment-motion effects on retention. They do not demonstrate that one sediment texture or nutrient regime is suitable across species and sites.

Initial rooting does not guarantee persistence. Pull tests of *Rhizophora mangle* and *Avicennia germinans* revealed age-dependent anchorage and removal modes; estimated uprooting velocities exceeded typical site flows, suggesting that erosion around established seedlings can precede their removal (Kibler et al., 2022). Cross-species experiments also found different responses to erosion and accretion (van Hespen et al., 2022). After establishment, local light can become particularly influential: a six-species cohort study found stronger associations of one-year survival with light than with hydrology in the investigated system (Sloey et al., 2022). These findings support a shift in limiting processes across stages rather than a single hierarchy of controls.

Planting bypasses propagule production, dispersal, and some settlement constraints, but planted seedlings still encounter unsuitable inundation, root-zone stress, and physical disturbance (Gorman et al., 2022). Natural recruitment, in contrast, records successful arrival and at least initial establishment, although later survival remains uncertain (Sousa et al., 2007; Sloey et al., 2022). The evidence therefore supports tracking arrival, retention, rooting, and persistence separately: a survival percentage alone cannot distinguish a shortage of viable propagules from failure after settlement.

## 3 Hydroperiod, elevation, and tidal connectivity establish the physical template for early mangrove establishment

Hydrology provides the physical template within which the stage-specific establishment filters described above operate. Tidal connectivity, surface elevation, microtopography, hydroperiod, and drainage jointly determine whether propagules are transported to a site, whether they are retained long enough to initiate roots, and whether the sediment environment remains suitable for early root function and seedling growth (Pérez-Ceballos et al., 2020; Van Loon et al., 2016). Hydroperiod should therefore be understood not simply as the presence or absence of water, but as the timing, frequency, duration, and depth of inundation and exposure, together with the capacity of water to enter, circulate through, and drain from a site (Pérez-Ceballos et al., 2020; Medina-Calderón et al., 2021). As synthesized in **Figure 2**, hydrological setting affects establishment through direct physical pathways, including propagule transport, retention, inundation exposure, and wave or current disturbance, and indirect root-zone pathways that influence salinity, oxygen availability, redox status, sulfide exposure, and root function. These controls must be interpreted relative to local tidal range, rainfall, freshwater inputs, geomorphic setting, sediment properties, and species or life-stage requirements rather than as fixed elevation or inundation thresholds.

Hydroperiod has non-linear and stage-dependent effects on early establishment. Periodic flooding may maintain sediment moisture, support propagule transport, and refresh porewater, whereas prolonged submergence or ineffective drainage can expose newly rooted seedlings to persistent saturation and oxygen limitation (Balke et al., 2011; Li et al., 2020). Conversely, severely reduced tidal exchange can limit propagule entry, reduce flushing, and promote evaporation-driven salt accumulation, causing local conditions to diverge from nearby open-water environments (Pérez-Ceballos et al., 2020). Experimental evidence illustrates this context dependence: *Avicennia marina* seedlings showed improved photosynthetic performance under moderate inundation but reduced performance under longer inundation, indicating that hydroperiod effects depend on duration rather than simply on flooding presence (Li et al., 2020). Other seedling studies similarly show that responses to inundation are modified by salinity, species identity, and growing conditions (Cardona-Olarte et al., 2006; Hoppe-Speer et al., 2011). Hydrological diagnosis should therefore focus on the form, duration, and seasonality of flooding rather than on a single water-level observation.

Elevation and microtopography determine how regional tidal forcing is expressed at the scale of propagules and roots. Surface position relative to the local tidal frame controls inundation frequency, drainage after high water, water residence time, sediment moisture, and the likelihood that propagules are retained or exported (Pérez-Ceballos et al., 2020; Van Loon et al., 2016). In low-relief intertidal landscapes, small elevation differences can generate sharply different establishment conditions because minor changes in surface height can alter the balance between flushing, ponding, drying, and sediment exposure. Recent restoration evidence shows that artificial changes in soil elevation can modify hydroperiod and sediment redox conditions together with seedling growth, indicating that elevation manipulation changes the broader root-zone environment rather than only the depth of standing water (Corona-Salto et al., 2025). Similarly, hydrological classification based on water-level records distinguished sites suitable for natural regeneration, suitable but unrecruited, and too wet for mangrove establishment, whereas elevation estimates alone could not resolve these alternatives (Van Loon et al., 2016). Elevation should therefore be treated as a locally calibrated indicator of hydrological exposure, not as a universal planting criterion.

Tidal connectivity determines whether hydrological exchange reaches the part of the landscape where establishment is expected to occur. Open and coherent tidal exchange can transport propagules, renew water, and support drainage, whereas restricted channels, silted creeks, impoundments, or poorly aligned connections can increase residence time and create ponded or poorly flushed areas (Pérez-Ceballos et al., 2020; Van Loon et al., 2016). The outcome depends on flow-path geometry, drainage gradient, and microtopographic routing, not merely on whether a channel is present. In an impaired Mexican mangrove wetland, rehabilitation guided by mapped preferential flow paths and microtopography produced more effective reconnection than channel placement based mainly on the shortest distance to a main tidal channel (Pérez-Ceballos et al., 2020). Linear excavated channels implemented without sufficient topographic information clogged rapidly and required greater maintenance, illustrating why simply opening a channel may not restore a suitable hydroperiod (Pérez-Ceballos et al., 2020). Hydrological rehabilitation should therefore aim to restore coherent flow paths, drainage gradients, and site-scale tidal exchange rather than only increasing water access.

The direct effects of the hydrological template are most evident before and during rooting. Tides and currents influence propagule dispersal, deposition, and retention, while wave exposure, erosion, and bed-level change can export recently settled propagules or uproot young seedlings before anchorage is secure (Balke et al., 2011; Van der Stocken et al., 2019). Hydrodynamic analyses and seedling pull tests further indicate that establishment depends on local waves, currents, surrounding vegetation, sediment properties, and species traits rather than on hydrological exposure alone (Cannon et al., 2020; Kibler et al., 2022). After roots enter the sediment, hydrology also acts indirectly by regulating saturation, residence time, flushing, and porewater exchange, thereby shaping the salinity and biogeochemical conditions experienced by roots (Medina-Calderón et al., 2021; Pérez-Ceballos et al., 2020). These linked pathways justify placing hydrology as the first diagnostic domain in restoration planning, while recognizing that it is not the only constraint controlling establishment.

Hydrological suitability is supported by measurements of inundation and drainage relative to functioning local reference stands, rather than by channel presence or elevation alone (Van Loon et al., 2016). The Mexican reconnection study shows how restoring flow paths can improve water exchange, but its engineering solution cannot be transferred without assessing local topography and sediment processes (Pérez-Ceballos et al., 2020). Thus, hydrological assessment informs intervention choice where exchange is impaired (Lewis, 2005), while persistent failure at a hydrologically compatible site requires investigation of other filters.

## 4 Porewater salinity and osmotic–ionic stress as a dynamic ecological filter

Porewater salinity is a dynamic ecological filter that links the hydrological template described above with early root function and seedling performance. It should not be interpreted as an independent or static site property because salinity regimes emerge from the interaction of tidal exchange, hydroperiod, evaporation, freshwater inputs, drainage, and sediment water residence time (**Figure 2**). Identical salinity values may therefore reflect different hydrological causes and have different implications for propagule retention, root initiation, and early seedling survival. Field and restoration studies show that tidal connectivity and water residence time regulate salinity distribution through flushing and accumulation processes, making salinity diagnosis dependent on hydrological context rather than on isolated measurements (Van Loon et al., 2016; Pérez-Ceballos et al., 2020). At the physiological level, elevated porewater salinity constrains early establishment through coupled osmotic and ionic stress. Higher salinity lowers porewater osmotic potential and can reduce water uptake unless plants maintain a sufficiently negative internal water potential through osmotic adjustment (Ball, 1988; Parida and Jha, 2010). Seedlings must also invest energy in ion exclusion, osmotic adjustment, and compartmentalization of Na^+^ and Cl^-^, which can increase respiratory demand and reduce carbon available for growth (Krauss et al., 2008; Parida and Jha, 2010). However, these responses vary among species and developmental stages because mangroves differ in their capacity for salt regulation, ion exclusion, and tissue-level tolerance (Ball, 1988). Salinity should therefore be interpreted as a stage- and species-dependent filter rather than as a single universal threshold.

Hydroperiod mediates salinity effects by controlling both salt input and salt removal. Periodic tidal flooding may flush salts from the root zone, whereas restricted exchange, prolonged residence time, poor drainage, or high evaporation can promote salinity accumulation. This pattern has been shown in restoration systems where hydrological reconnection reduced porewater salinity through improved water exchange (Pérez-Ceballos et al., 2020). Conversely, poorly drained sites may accumulate more salts even when external water salinity is not extreme, indicating that the structure of flooding and drainage can be more important than seawater concentration alone. Experimental evidence also shows that salinity and flooding can interact to produce non-linear growth responses in mangrove seedlings, reinforcing the need to evaluate salinity as part of a coupled hydroperiod–root-zone system (Hoppe-Speer et al., 2011).

Salinity and oxygen limitation can co-occur where exchange and drainage are impaired, but their covariance is site-specific. Experimental redox treatments affect seedling carbon assimilation and allocation, whereas field reconnection can alter salinity and sulfide together (Pezeshki et al., 1997; Pérez-Ceballos et al., 2020). These evidence types support examining interacting root-zone stresses; they do not establish a universal synergistic effect of salinity and sulfide. Direct factorial tests are needed to distinguish additive effects, interactions, and shared hydrological causes (Lyimo and Mushi, 2005; Hu et al., 2025). Salinity alone is therefore an unreliable predictor of establishment success. Mangrove responses to salinity are modified by species identity, seedling age, exposure duration, and co-occurring flooding conditions, producing substantial variation even under apparently similar salinity regimes (Ball, 1988; Pezeshki et al., 1997; Kodikara et al., 2018). This helps explain why seedlings may fail under apparently “moderate” salinity when drainage, flushing, inundation timing, or sediment redox conditions remain unsuitable. In restoration planning, decisions based on single-point salinity measurements risk overlooking the temporal and spatial variability that seedlings actually experience.

Spatially patchy survival does not identify salinity as the sole cause: propagule delivery, retention, light, and physical exposure also vary among microsites (Sousa et al., 2007; Sloey et al., 2022; Smith et al., 2022). Measurements of porewater salinity should therefore be paired with water level and drainage observations, as illustrated by hydrological restoration studies (Pérez-Ceballos et al., 2020). Repeated observations at comparable rooting depths and across seasons are a proposed diagnostic extension of this evidence, not a validated sampling rule for every setting. Collectively, salinity experiments and restoration observations support interpreting exposure duration, developmental stage, and hydrological context alongside salinity magnitude (Kodikara et al., 2018; Pérez-Ceballos et al., 2020; Kimera et al., 2024). Salinity measurements alone cannot establish whether poor growth reflects osmotic and ionic demands, impaired root aeration, or another co-occurring limitation. **Table 2** therefore treats salinity accumulation as a hypothesis requiring confirmation, with alternative explanations retained.

## 5 Redox potential, sulfide toxicity, and anaerobic impairment of root-zone function

Hydrological conditions influence early mangrove establishment not only by controlling inundation exposure, but also by regulating sediment oxygen availability and redox status. Hydroperiod, drainage, water residence time, sediment texture, organic-matter supply, and microbial oxygen demand together determine whether the rooting zone remains sufficiently oxidized for root initiation and seedling growth growth (Pezeshki and DeLaune, 2012; Van Loon et al., 2016). Sediment saturation can promote reducing conditions, but saturation and redox state are not equivalent. The intensity and duration of reduction depend on organic substrates, sediment structure, tidal exchange, root activity, and local geomorphology (Pezeshki and DeLaune, 2012; Pérez-Ceballos et al., 2020). Redox potential should therefore be treated as a dynamic root-zone property rather than as a fixed characteristic of wet sediment.

As oxygen becomes depleted, microbial metabolism shifts from aerobic respiration toward alternative electron-accepting pathways, and sulfate reduction may become important in sulfate-rich coastal sediments (Pezeshki and DeLaune, 2012; Pérez-Ceballos et al., 2022). This process can generate dissolved sulfide, but sulfide concentration is not controlled by sulfate reduction alone. It also reflects oxidation, sediment geochemistry, diffusion, tidal exchange, water residence time, and biological modification of the rhizosphere (McKee et al., 1988; Pérez-Ceballos et al., 2022). Redox decline and sulfide exposure are therefore related but not identical constraints. A low redox environment may impair root function even before sulfide reaches toxic concentrations, whereas sulfide accumulation can add a more specific chemical stress to already oxygen-limited roots.

Foundational experiments demonstrate mechanisms that remain relevant even though current mangrove conditions differ among sites. Hypoxia altered root metabolism in *Avicennia germinans*, and subsequent experiments found species-specific physiological responses and changes in carbon assimilation and allocation under low-redox treatments (McKee and Mendelssohn, 1987; McKee, 1996; Pezeshki et al., 1997). These studies establish physiological plausibility, not present-day exposure levels or universal tolerance limits. More recent work links radial oxygen loss to rhizosphere nitrification and nitrogen acquisition (Cheng et al., 2020), while inundation experiments show physiological and proteomic adjustment in *Avicennia marina* seedlings (Li et al., 2020). Root aeration capacity and exposure regime must therefore be considered together.

Lyimo and Mushi (2005) combined observations of mangrove sediments in Dar es Salaam, Tanzania, with a glasshouse establishment experiment involving *Avicennia marina* and *Rhizophora mucronata*. Their results linked reduced, sulfidic conditions with establishment constraints and documented contrasts between rooted and root-free sediment. The classic Belize study likewise found altered sulfide and redox conditions near aerial roots (McKee et al., 1988). More recent field work examined sulfide in tide-dominated mangrove degradation (Pérez-Ceballos et al., 2022). The latter concerns ecosystem degradation and provides contextual evidence, rather than a transferable toxicity threshold for newly rooted seedlings.

Hydrological intervention provides complementary field evidence: flow-path reconnection in an impaired Mexican mangrove changed salinity and sulfide concurrently (Pérez-Ceballos et al., 2020). Artificial elevation treatments also altered hydroperiod and redox conditions in a restored Mexican mangrove (Corona-Salto et al., 2025). These cases support evaluating water exchange and rooting-zone chemistry together, but neither isolates all physiological interactions among salinity, hypoxia, and sulfide. Earlier flooding and salinity experiments provide mechanistic context (Pezeshki et al., 1990); field covariation alone cannot establish the relative contribution of each stressor.

Redox conditions vary with depth, roots, tidal stage, and biological activity (Lyimo and Mushi, 2005; Cheng et al., 2020). Recent burrow studies add evidence that this variation is not controlled by roots alone: fiddler crabs alter sediment microbial niches (Booth et al., 2019), and burrows in colonizing French Guiana mudflats exchange solutes with tidal water and can become anoxic when irrigation stops (Aller et al., 2024). These studies concern sediment processes, not controlled tests of seedling survival. They explain why a bulk redox reading may fail to characterize exposure at a young root surface; the aeration capacity of recently rooted seedlings remains a specific research gap.

The synthesis distinguishes oxygen limitation, redox state, and sulfide exposure rather than treating them as interchangeable measurements. Persistent reduction can be natural in mangrove sediments; its diagnostic significance depends on plant response, depth, timing, and reference conditions (Pezeshki and DeLaune, 2012; Pérez-Ceballos et al., 2022). Hydrological repair is a candidate response when impaired exchange is demonstrated (Van Loon et al., 2016; Pérez-Ceballos et al., 2020), not a prescription to drain all anoxic sediment. **Table 2** pairs chemical and hydrological observations with competing explanations before an intervention is selected.

## 6 Sediment instability, erosion, burial, and hydrodynamic forcing

Sediment dynamics impose a physical establishment filter that is distinct from, but often coupled to, the hydro-biogeochemical constraints described above. Propagules and young seedlings establish in mobile intertidal substrates where waves, currents, drag, scour, erosion, accretion, burial, and bed-level change can determine whether they remain in place long enough to develop functional roots. Compatible root-zone chemistry does not guarantee establishment if disturbance prevents retention or anchorage (Balke et al., 2011; Kibler et al., 2022). Experimental and modelling studies show that both the magnitude and sequence of erosion and accretion events can influence seedling survival, morphology, and later resistance to disturbance (Balke et al., 2013b; Le Minor et al., 2019). Sediment instability should therefore be treated as a primary diagnostic domain rather than as a secondary consequence of hydrology.

The first mechanical bottleneck occurs between propagule arrival and secure anchorage. Water and wind influence propagule transport before settlement, while local elevation, tidal flow, surface roughness, and microsite structure determine whether propagules are retained or exported (Van Der Stocken et al., 2015; Wang et al., 2019). In a Louisiana saltmarsh–mangrove ecotone, fewer than 10% of marked *Avicennia germinans* propagules were retained within the study area, whereas most were exported, demonstrating that propagule supply and successful retention are separate processes (Yando et al., 2021). Experimental work with *Avicennia alba* further showed that early establishment requires a temporary low-disturbance window during which roots can develop before later erosion, drag, or wave forcing occurs (Balke et al., 2011). Sheltered settings can therefore support establishment where exposed areas fail, although the strength of this effect depends on species, propagule traits, sediment properties, and local hydrodynamic conditions (Thampanya et al., 2002a).

After rooting begins, erosion can limit establishment through several pathways. Direct drag may remove recently rooted seedlings, whereas gradual scour around the stem base can reduce root support and increase vulnerability to toppling or uprooting during later high-energy events (Balke et al., 2013b; Le Minor et al., 2019). Field pull tests and laboratory experiments with *Rhizophora mangle* and *Avicennia germinans* showed that newly established seedlings mainly failed through root pull-out, while older seedlings more often failed through root breakage as root systems developed (Kibler et al., 2022). Surrounding vegetation increased anchoring force in the same study, indicating that seedling persistence depends not only on individual root development but also on the local structural setting (Kibler et al., 2022). These experiments support a mechanical explanation for age-dependent loss; however, species-specific critical flow velocities should not be transferred directly among sites because anchorage depends on seedling age, root architecture, sediment cohesion, vegetation density, microsite shelter, and the timing and magnitude of disturbance events.

Accretion and burial are not simply the opposite of erosion. Moderate and gradual sediment addition may be tolerated by some seedlings, but repeated, rapid, or deep burial can reduce survival, alter growth, or overwhelm early root and shoot development. In burial experiments, mangrove species differed in survival and growth responses to increasing sediment accretion, indicating that burial tolerance depends on species traits, plant size, sediment type, and disturbance history (Thampanya et al., 2002b). The dynamic-sediment experiment of Balke et al. (2013b) further showed that cumulative erosion and accretion can influence both seedling morphology and the amount of later disturbance that seedlings can withstand. Event-driven disturbance may be especially damaging; in a southern Red Sea field study, sandstorms were associated with substantial mortality and reduced growth of *Avicennia marina* seedlings (Abrogueña et al., 2022). These findings argue against applying a universal erosion or burial threshold to restoration planning.

Sediment texture and cohesion shape the mechanical environment in which roots develop and persist. Sediment texture influences root-system development, while sediment strength and root anchorage affect resistance to displacement (He et al., 2022; Kibler et al., 2022). A mesocosm experiment with *Kandelia obovata* and *Sonneratia apetala* showed that root morphological traits differed strongly among sediment-texture treatments, and that species responded differently to substrate conditions (He et al., 2022). These results show that nutrient responses depend on substrate and species; they do not prove that nutrient addition is ineffective in every physically stressful setting. Sediment texture should therefore be interpreted together with hydrodynamic exposure, bed-level change, elevation, drainage, and the expected rooting strategy of the target species.

Species traits, developmental stage, and microsite structure modify the physical filter. Across eight mangrove species, experimentally imposed erosion and accretion produced contrasting survival, root elongation, toppling resistance, and dislodgement responses, reflecting differences in propagule size, root development, and growth strategy (van Hespen et al., 2022). Microsite features can also alter local retention and exposure. In Florida saltmarsh–mangrove ecotones, resident vegetation retained more range-expanding mangrove propagules than other structures during deeper spring tides in interior habitats, whereas propagules were largely lost from exposed fringe habitats regardless of local structure (Smith et al., 2022). These findings support the diagnostic use of naturally sheltered depressions, vegetation patches, woody debris, root mats, and other retentive microsites as indicators of locations where physical establishment may be feasible.

Physical disturbance acts before and after rooting: propagules may be exported, while rooted seedlings can be dislodged, scoured, or buried (Balke et al., 2011, 2013b; Kibler et al., 2022). Species differences in these responses support measuring bed-level change and anchorage alongside seedling fate instead of transferring a single erosion or burial threshold (van Hespen et al., 2022). Protecting shelter or restoring sediment processes is a conditional management inference from these studies; local measurements must establish whether it addresses the actual cause of loss (Lewis, 2005; Sousa et al., 2007).

## 7 Species-, life-stage-, and microsite-dependent responses to interacting establishment stressors

The hydroperiod, salinity, reducing sediment conditions, sediment instability, and hydrodynamic processes discussed above do not act on a uniform biological target. Establishment outcomes depend on species identity, developmental stage, exposure sequence, and centimetre- to metre-scale microsite conditions (Sloey et al., 2022; Hu et al., 2025). A site may retain or support propagules of one taxon or life stage while constraining another under the same broad tidal regime. Experiments involving salinity, flooding, competition, and sediment dynamics consistently show responses to interacting stressors rather than to single, universally transferable tolerance thresholds (van Hespen et al., 2022; Kimera et al., 2024; Hu et al., 2025). **Figure 3** therefore presents study-linked examples of trait responses, developmental constraints, and microsite modifiers as conceptual guides, not fixed rankings for all members of a genus.

Species differ in propagule form, root-development strategy, salt-management traits, early growth rate, and biomass allocation (He et al., 2022; Kimera et al., 2024). These differences influence how propagules and seedlings respond to the same environmental setting. In a controlled salinity study, *Avicennia marina* and *Rhizophora mucronata* differed in emergence, growth, survival, and mineral-element responses across freshwater, diluted seawater, seawater, and brine treatments (Kimera et al., 2024). Root-system morphology of *Kandelia obovata* and *Sonneratia apetala* also responded differently to sediment texture, showing that substrate conditions can interact with species-specific root-development strategies (He et al., 2022). Across eight mangrove species, experimentally imposed erosion and accretion produced contrasting survival, shoot, and root responses related to trait differences and disturbance regime (van Hespen et al., 2022). These studies support species–microsite matching, but they do not justify global rankings of salinity, flooding, or sediment-dynamics tolerance.

Life stage changes both exposure and response capacity. Depending on the taxon, the earliest establishment transition may involve propagule delivery, settlement, retention, root initiation, and anchorage rather than conventional seed germination. Propagules and recently rooted seedlings can be lost before physiological tolerance is fully expressed because root biomass, anchorage strength, and rhizosphere modification capacity are still limited (Balke et al., 2011; Kibler et al., 2022). In pull tests, recently established *Rhizophora mangle* and *Avicennia germinans* seedlings primarily failed through root pull-out, whereas older seedlings more often failed through root breakage; surrounding vegetation also increased anchoring force in the reported field settings (Kibler et al., 2022). Salinity responses can also shift with age, as shown in a 30-week seedling experiment where growth and survival responses changed across developmental stages (Kodikara et al., 2018). Later seedlings and saplings may have greater anchorage and stronger root-zone modification capacity, but persistent salinity, reduced sediment, burial, erosion, or competition can still constrain performance.

Microsites modify these processes over short distances. Small differences in elevation, drainage, surface roughness, sediment cohesion, vegetation cover, woody debris, root mats, light availability, and local hydrodynamic shelter can alter propagule retention, root-zone conditions, sediment stability, and seedling survival (Sloey et al., 2022; Smith et al., 2022). In a saltmarsh–mangrove ecotone, resident vegetation retained more range-expanding mangrove propagules than other local structures during deeper spring tides, whereas propagules were largely lost from exposed fringe sites (Smith et al., 2022). A six-species Southeast Asian cohort further showed that one-year seedling survival was associated with local abiotic conditions in species-specific ways (Sloey et al., 2022). After hydrological rehabilitation in a Mexican lagoon, pioneer nurse vegetation facilitated mangrove recruitment, but this facilitation followed restoration of water exchange and should not be interpreted as replacing hydrological correction (Teutli-Hernández et al., 2019). Nurse vegetation, debris, and root mats can therefore create local establishment windows, but shading, competition, sediment trapping, or herbivory may offset these benefits in other settings.

Interactions among stressors are especially important where a species performs well under one constraint but fails under another. A species with comparatively strong salinity tolerance may still fail where propagules are exported before rooting, where sediment instability prevents anchorage, or where prolonged ponding creates reducing root-zone conditions. Likewise, a species with a robust root system in a sediment experiment may remain unsuitable where restricted tidal exchange produces salinity accumulation or sulfide exposure (He et al., 2022; Pérez-Ceballos et al., 2020). A factorial juvenile-mangrove study showed that salinity, flooding, and aboveground competition jointly influenced biomass and allometric responses, reinforcing that establishment outcomes depend on stressor combinations rather than isolated tolerance traits (Hu et al., 2025). The objective is therefore not to identify a universally “tolerant” species, but to determine which stressor combination prevents the relevant establishment transition at a given site.

Species–microsite matching requires evidence on establishment, not adult zonation alone. Seed sources, propagule arrival, local substrate, and light conditions help distinguish limited supply from post-arrival failure (Sousa et al., 2007; Sloey et al., 2022; Smith et al., 2022). Rehabilitation principles support correcting confirmed site constraints before enrichment planting (Lewis, 2005; Pérez-Ceballos et al., 2020), but competition and other biotic feedbacks may remain important after physical conditions improve. **Table 1** summarizes the interacting mechanisms and their illustrative evidence.

Vegetation can both create and close establishment opportunities. Existing plants can trap propagules and shelter seedlings, whereas dense competitors reduce available light and space and can change sediment retention (Teutli-Hernández et al., 2019; Smith et al., 2022). In a Chinese estuary experiment, invasive *Spartina alterniflora* suppressed growth of native *Kandelia obovata* and introduced *Sonneratia apetala*; responses depended on elevation, and the introduced mangrove later escaped the grass canopy more readily (Peng et al., 2018). This illustrates why native nurse vegetation and invasive competitors cannot be assigned the same management role. Vegetation identity, canopy light, propagule interception, and sediment change should be assessed together rather than assuming that greater plant cover always facilitates recruitment.

Crabs couple physical sediment reworking with biogeochemical and trophic effects. Burrow-associated microbial and redox conditions differ from bulk sediment (Booth et al., 2019), and tidally irrigated burrows can transport oxygenated water and nutrients into colonizing mudflats, although they may become anoxic during exposure (Aller et al., 2024). Excavation can also disturb settlement surfaces, while herbivorous crabs consume propagules in species- and habitat-dependent ways (Van Nedervelde et al., 2015). Improved aeration therefore does not establish a net survival benefit. Burrow activity, local sediment displacement, and propagule damage are complementary observations needed to distinguish facilitation from physical disturbance or predation.

Plant-associated microbes add further context dependence. Field tracer experiments demonstrate nitrogen fixation associated with *Avicennia marina* roots (Inoue et al., 2024), providing a mechanism for nutrient acquisition but not a direct test of newly settled propagules. Pot experiments found mycorrhizal colonization in *Bruguiera gymnorhiza* but not *Rhizophora stylosa* (Akaji et al., 2024), cautioning against assuming a common fungal association across mangroves. A seedling inoculation experiment showed that foliar endophytes could improve *Rhizophora mangle* performance under salinity, with benefits depending on the inoculum’s salinity history (Subedi et al., 2022). That result concerns leaf-associated microbes, not rhizosphere colonization; oxidative-stress buffering remains a possible mechanism rather than a demonstrated general explanation. Together with root oxygen–nitrogen coupling (Cheng et al., 2020), these findings support testing microbial contributions to establishment without prescribing unvalidated inoculation as a substitute for suitable habitat.

## 8 A diagnosis-first restoration framework for early mangrove establishment failure

Poor recruitment, mortality, and stunting can arise from different establishment filters and cannot, by themselves, identify a planting deficit (Gorman et al., 2022; Zimmer et al., 2022). The proposed framework builds on ecological rehabilitation and adaptive management (Lewis, 2005; Ellison et al., 2020) and on the mechanisms synthesized in sections 2–7. Its novelty is the explicit connection between a failed life-stage transition, competing causal explanations, measurements, and a conditional response. **Figure 4** presents this as an iterative assessment, and **Table 2** identifies sources for each diagnostic domain. Neither the full sequence nor its intervention priorities have been validated in a comparative multi-site trial.

**Figure 4:**
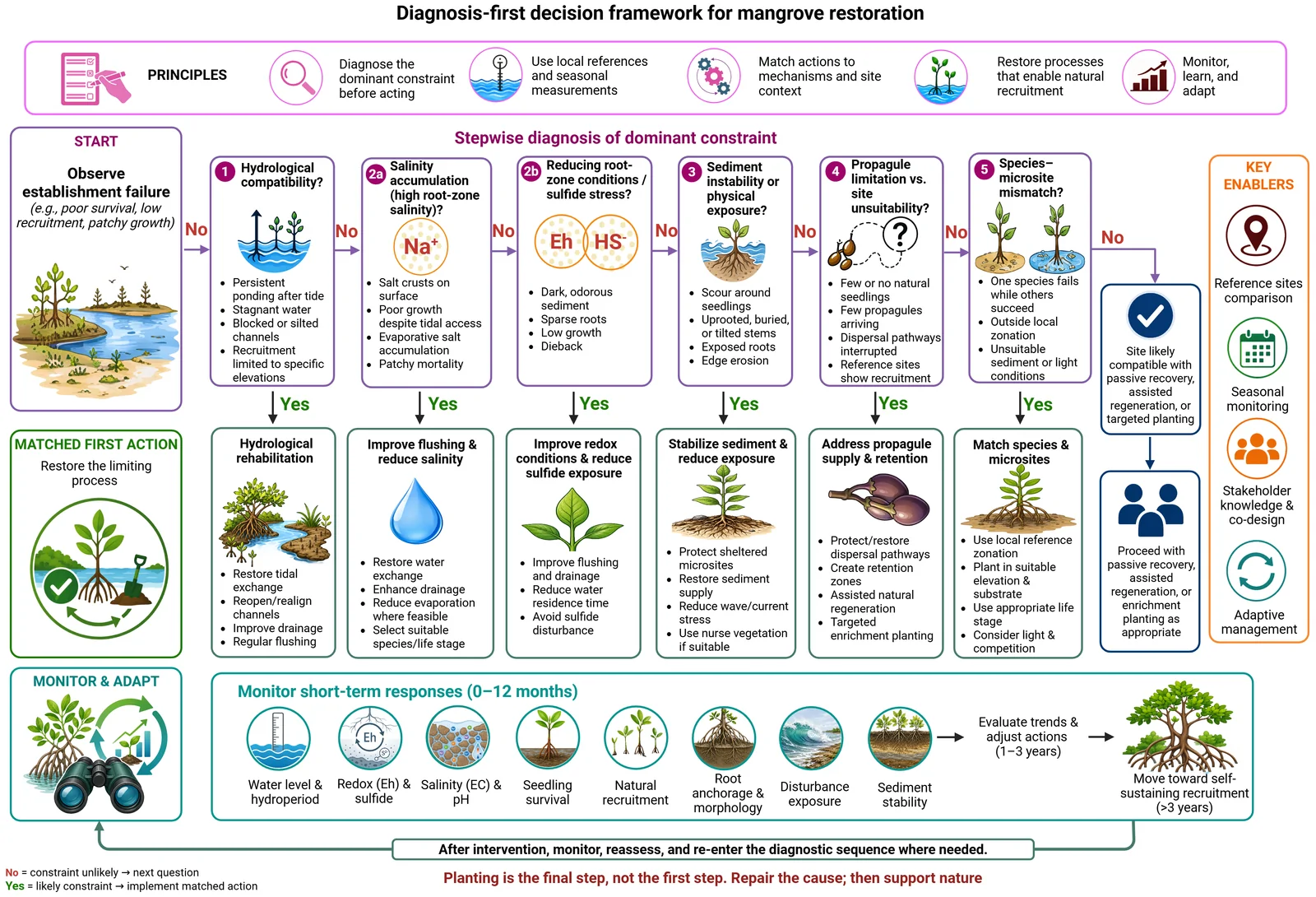
An iterative diagnosis-first assessment for mangrove restoration. The proposed pathway translates the evidence in Sections 2–7 and **Table 2** into candidate explanations, confirmation measurements, and conditional actions, building on process-based rehabilitation and adaptive management (Lewis, 2005; Ellison et al., 2020; Zimmer et al., 2022). Hydrological incompatibility is assessed as a possible constraint, alongside chemical, physical, supply, and biotic constraints. Supported findings justify evaluating a matched response; adequately assessed but unsupported explanations redirect assessment; unresolved evidence requires more observations. Concurrent constraints can require coordinated action. Monitoring tests the predicted establishment response and feeds back to diagnosis. The complete pathway is an author-developed conceptual synthesis, not a validated algorithm or a guarantee of recovery within a prescribed period.

Each candidate constraint should be classified as supported, not supported by adequate observations, or unresolved. An unresolved case requires further measurement; it must not be treated as absence of a constraint. Several supported constraints may require coordinated action because hydrology, chemistry, sediment dynamics, and biotic interactions can co-occur (Van Loon et al., 2016; Pérez-Ceballos et al., 2020). Following intervention, the limiting transition may change, requiring reassessment (Ellison et al., 2020; Zimmer et al., 2022). The framework therefore uses field symptoms to prioritize observations, not as stand-alone evidence for engineering or planting.

Hydrology is an initial organizing assessment because water exchange influences both delivery and root-zone exposure, as demonstrated by hydrological classification and reconnection studies (Van Loon et al., 2016; Pérez-Ceballos et al., 2020). This ordering is pragmatic, not a universal causal hierarchy. Where hydroperiod is compatible with local reference conditions, propagule limitation, exposure, light, or herbivory may deserve immediate attention (Sousa et al., 2007; Sloey et al., 2022). Natural basin ponding must be distinguished from anthropogenic restriction before changing channels or elevation. Rehabilitation is justified by demonstrated impairment and an explicit recovery objective, not by the presence of waterlogged sediment alone (Lewis, 2005).

After hydrological compatibility is evaluated, diagnosis should determine whether salinity accumulation, reducing root-zone conditions, sulfide exposure, or sediment instability is preventing establishment. These constraints may arise together but require separate confirmation because their visible symptoms can overlap. Salt crusts and stunted growth may indicate evaporation-driven salinity accumulation or poor flushing, but they may also reflect seasonal salinity pulses, unsuitable drainage, or interactions with sediment redox conditions (Pérez-Ceballos et al., 2020, 2022; Kimera et al., 2024). Black or odorous sediment, sparse roots, and early dieback may indicate persistent reducing conditions or sulfide exposure, but dark organic-rich sediment alone does not prove that the root zone is chemically unsuitable (Lyimo and Mushi, 2005; Dittmann et al., 2022; Pérez-Ceballos et al., 2022). Scour, burial, tilted stems, exposed roots, and post-storm seedling loss may indicate physical instability, but herbivory, failed rooting, propagule export, or weak anchorage can produce similar outcomes (Balke et al., 2011; Dittmann et al., 2022; Kibler et al., 2022). Diagnosis should therefore integrate porewater salinity or EC, redox potential, sulfide where feasible, sediment texture, bed-level change, hydrodynamic exposure, root condition, and seedling response across tidal and seasonal variation.

Absence of natural regeneration should not automatically be interpreted as evidence that the site is unsuitable. Recruitment failure can result from limited source populations, interrupted dispersal pathways, propagule export, weak retention, unsuitable rooting conditions, or mortality soon after arrival (Sousa et al., 2007; Balke et al., 2011). Distinguishing between these alternatives is essential because the appropriate restoration response differs. A site with suitable hydrology, sediment condition, and rooting environment but limited propagule arrival may benefit from restored dispersal pathways, retention features, assisted natural regeneration, or carefully targeted enrichment planting. In contrast, a site that receives propagules but loses them through export, burial, or early root failure requires correction of retention, sediment stability, or root-zone constraints before planting is justified (Balke et al., 2011; Smith et al., 2022). Species–microsite mismatch should be assessed after these broader site-process constraints have been evaluated. Local reference stands, nearby seed sources, seasonal propagule arrival, naturally recruited patches, elevation position, sediment condition, light environment, and disturbance history can guide species and life-stage selection (Sloey et al., 2022; Smith et al., 2022). Species selection must account for residual stress and cannot be assumed to remedy restricted water exchange, continuing erosion, or missing propagule pathways (Lewis, 2005; He et al., 2022; Zimmer et al., 2022).

Interventions are hypotheses about recovery pathways, not guaranteed remedies. Restoring flow paths may address impaired exchange (Pérez-Ceballos et al., 2020); protecting sheltered settlement zones may address mechanical loss (Kibler et al., 2022; van Hespen et al., 2022); and targeted planting may address limited arrival at an otherwise suitable site (Sousa et al., 2007; Zimmer et al., 2022). Species and developmental stage should be matched to verified exposure conditions (Kodikara et al., 2018; He et al., 2022; Hu et al., 2025). Competition, herbivory, and microbial processes modify these responses (Section 7.1) and should be assessed alongside abiotic constraints. In particular, improved sediment aeration need not offset propagule consumption, and nurse effects need not outweigh invasive competition.

**Table 2** links each diagnostic domain to field cues, candidate mechanisms, confirmation measurements, alternatives, and conditional first actions. Its source column distinguishes component evidence from the authors’ proposed translation into management; no cited study validates every step or action in a row. Repeated measurements, reference-site comparisons, and intervention monitoring provide tests of those proposals (Van Loon et al., 2016; Ellison et al., 2020). Follow-up should track arrival, retention, rooting, survival, and growth across relevant seasons and disturbances (Balke et al., 2011; Gorman et al., 2022; Sloey et al., 2022). If an intervention changes the presumed stressor without improving the predicted establishment transition, the diagnosis should be reconsidered. The framework’s effectiveness remains to be evaluated using replicated interventions and suitable comparisons.

## 9 Knowledge gaps and future research directions

The diagnosis-first framework developed in this review provides a structured basis for identifying likely constraints on early mangrove establishment, but diagnosis and prediction are not equivalent. Diagnosis tests candidate explanations for establishment failure at a specific site, whereas prediction requires estimating the probability of successful establishment before restoration actions are implemented. Global syntheses show that planted-mangrove survival varies with environmental setting, species, restoration age, and methodological decisions, highlighting the limitations of planting-centred prediction when site processes are not resolved (Gorman et al., 2022). Predictive restoration therefore requires evidence that links hydrological exposure, root-zone chemistry, sediment dynamics, propagule processes, biological transitions, seasonal variability, and disturbance history across scales, rather than relying on single surveys, short experiments, or isolated tolerance measurements.

A major limitation is the scarcity of integrated, stage-resolved monitoring datasets. Few studies jointly and repeatedly measure water level, hydroperiod, porewater salinity, redox potential, sulfide exposure, sediment change, propagule arrival and retention, rooting success, seedling survival, and early growth within the same restoration landscape. Existing studies provide important components of this evidence base, including hydrological classification of restoration suitability (Van Loon et al., 2016), flow-path-based hydrological reconnection linked with changes in salinity and sulfide (Pérez-Ceballos et al., 2020), salinity responses of mangrove seedlings (Kimera et al., 2024), hydroperiod–salinity effects on root dynamics (Medina-Calderón et al., 2021), and age-dependent anchorage failure under hydrodynamic loading (Kibler et al., 2022). The next step is to connect these measurements within coordinated field designs that follow tagged propagules or seedlings from arrival and retention through rooting, survival, and transition toward sapling establishment.

A second gap is the scale mismatch between controlled experiments and restoration-scale field environments. Laboratory and mesocosm studies can isolate mechanisms such as salinity tolerance, sediment-texture effects, root development, erosion resistance, and burial response, but they cannot fully reproduce fluctuating tides, propagule transport, sediment mobility, hydrodynamic exposure, and disturbance sequences in intertidal landscapes (Van Loon et al., 2016; Pérez-Ceballos et al., 2020; He et al., 2022; Kimera et al., 2024). Conversely, field studies capture environmental realism but often lack the fine-scale measurements needed to identify causal pathways. Future work should therefore pair experimental control with field validation by combining replicated restoration plots, local reference stands, continuous water-level monitoring, repeated porewater and sediment measurements, and stage-specific seedling observations across contrasting tidal, geomorphic, and climatic settings.

Mechanistic uncertainty remains high because interacting stressors are rarely measured together. Hydroperiod, salinity, redox potential, sulfide exposure, sediment instability, propagule limitation, species traits, and microsite conditions are often studied separately, yet establishment failure commonly emerges from their combined effects. Restricted hydrological exchange, for example, can simultaneously promote salinity accumulation, prolonged saturation, redox decline, and sulfide exposure, while sediment instability can prevent anchorage before physiological tolerance is expressed (Balke et al., 2011; Dittmann et al., 2022; Kibler et al., 2022; Pérez-Ceballos et al., 2022). These interactions make universal thresholds for salinity, elevation, inundation duration, redox potential, sulfide concentration, sediment stability, or seedling size unlikely to be transferable across sites. Future studies should instead quantify response gradients, exposure duration, recovery capacity, interaction effects, and stage-specific transition probabilities.

Longer-term and disturbance-sensitive monitoring is also needed. Short-term seedling survival is useful but insufficient because early establishment depends on persistence through tidal cycles, seasonal salinity shifts, erosion or accretion events, storms, droughts, herbivory, and competition. Monitoring should extend beyond initial planting or recruitment surveys to track root development, anchorage, growth, sediment surface change, natural recruitment, and transition toward sapling stages. Such monitoring would help distinguish sites suitable for passive recovery from those requiring hydrological repair, sediment stabilization, assisted natural regeneration, or targeted enrichment planting.

Spatial technologies can document topography, canopy structure, shoreline change, and recruitment patterns, but they cannot directly resolve all root-zone mechanisms (Beselly et al., 2021; Xie et al., 2022). Recent UAV work demonstrates mangrove seedling detection while also identifying limitations caused by survey conditions and small plant size (Shendryk and Vilas, 2026a; see correction, Shendryk and Vilas, 2026b). Such observations require field calibration for porewater salinity, sulfide, root function, and propagule retention. Predictive models additionally need independent validation that accounts for spatial and temporal dependence (Roberts et al., 2017). Remote sensing and modelling thus complement stage-resolved measurements rather than replace them.

The studies assembled in this narrative synthesis do not validate the combined diagnostic pathway proposed here; this is not an exhaustive claim that no integrative model exists. A useful predictive framework would need to combine process-based hydrological and sediment information with biological response functions, stage-specific transition probabilities, local reference conditions, and explicit uncertainty. Testing predictive uses of the diagnosis-first pathway will require that diagnostic cues, confirmation measurements, interventions, and biological outcomes are recorded consistently across projects. Progress will depend on converting site diagnosis from a one-time assessment into a validated, uncertainty-aware prediction system that identifies likely establishment bottlenecks before restoration action is taken.

## 10 Conclusion

Early mangrove establishment depends on successful passage through delivery, retention, rooting, and persistence constraints whose importance varies with setting, species, and developmental stage. The reviewed evidence supports distinguishing propagule limitation from post-arrival mortality and examining hydrology, root-zone chemistry, sediment dynamics, and biotic modifiers together. The proposed diagnosis-first framework makes these connections explicit through candidate mechanisms, confirmation measurements, and conditional actions. It provides a transparent basis for testing restoration decisions but does not supply universal tolerance thresholds or demonstrate superior restoration performance. Where physical or chemical impairment is confirmed, process rehabilitation may precede planting; where suitable habitat lacks propagules, assisted regeneration may be appropriate. Local reference conditions, repeated observations, and follow-up of life-stage transitions are necessary to assess whether the selected action improves establishment and eventual self-sustaining recruitment.
